# A next-generation prediction risk model for acute myocardial infarction: Derivation and validation in a multi-centre cohort

**DOI:** 10.1016/j.ijcrp.2026.200659

**Published:** 2026-06-08

**Authors:** Jose David Amorocho-Morales, Sergio Parra Guevara, Elias Quintero-Muñoz, Guillermo Dimas, Juan Esteban Correa-Morales

**Affiliations:** a Universidad de La Sabana, Chía, Colombia; b Universidad Simón Bolívar, Cúcuta, Colombia; c Salud Total, Bogotá DC, Colombia; d Faculty of Medicine and Health Sciences, UIC Barcelona – Universitat Internacional de Catalunya, Sant Cugat Del Vallès, Barcelona, Spain

**Keywords:** Acute myocardial infarction, Risk prediction model, Machine learning in cardiology, Short-term cardiovascular risk, Population health stratification

## Abstract

**Aim:**

Cardiovascular disease remains the leading global cause of death, and the need for accurate, event-specific risk prediction is particularly critical in regions where long-horizon models perform poorly. We developed and internally validated a probabilistic model to estimate 6- and 12-month risk of acute myocardial infarction, with exploratory 5- and 10-year horizons, using routinely collected electronic health record data from an integrated cardiovascular cohort in Colombia.

**Methods:**

The study followed TRIPOD + AI guidance and analysed 382,589 patients contributing 3.9 million encounters. The modelling strategy combined a calibrated gradient-boosting classifier with an interpretable survival ensemble incorporating Cox regression, random survival forests, and discrete-time hazards. Primary outcomes were prediction accuracy, discrimination, calibration, and concordance with legacy score scales.

**Results:**

The classifier achieved an AUC of 0.869, while 6- and 12-month survival models reached C-indices of 0.836 and 0.846. Calibration was strong, with predicted vs observed AMI counts nearly identical (O/E = 0.998). Concordance analyses demonstrated only moderate alignment with Framingham and PROCAM, indicating substantial re-ranking at short horizons compared with legacy long-term models. External, label-delayed validation (n = 5602) showed monotonic risk separation across predefined priority bands.

**Conclusion:**

This model provides a practical population-health stratification tool for short-term AMI risk, with particular value in resource-constrained settings. Recalibration to local incidence rates is recommended before deployment. Prospective evaluation is warranted to assess real-world clinical and operational impact.

## Highlights

• ML model using EHR data predicted 6- and 12-month AMI risk (AUC 0.869; C-index 0.836–0.846; O/E=0.998) in 382,589 patients.
• Risk probabilities stratified into priority bands enable identification and prioritization of patients at highest near-term AMI risk.
• The model accurately identified high-risk patients often missed or differently assessed by traditional long-term scoring systems.

## Graphical abstract

**Figure:**
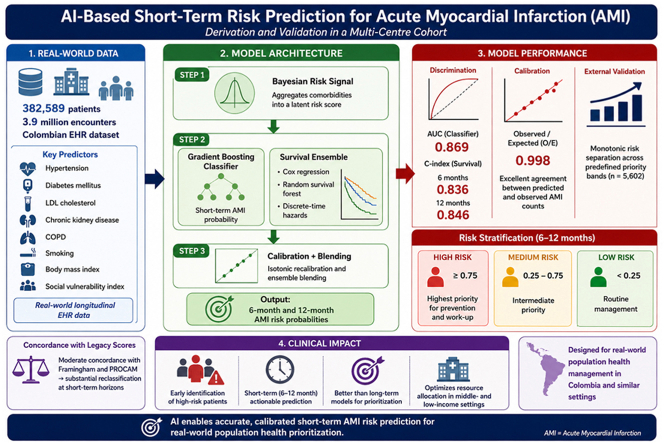

## Teaser

Lay summary.

## 1 Introduction

Cardiovascular diseases (CVD) remain the leading cause of morbidity and mortality worldwide, representing one of the greatest global health and economic burdens [1].In 2023, CVD caused roughly 19.2 million deaths, most of which were attributable to ischemic heart disease, the main cause of heart attacks [2]. Moreover, heart attacks contributed to 437 million disability-adjusted life years in 2023 [2]. Coronary heart disease, including acute myocardial infarction (AMI), is estimated to affect over 250 million people globally, reflecting the growing prevalence and chronicity of cardiovascular conditions [3]. In response to evolving epidemiological patterns and the limitations of traditional cardiovascular risk models, newer and more comprehensive approaches to risk prediction have emerged [4].

The 2025 ACC/AHA guidelines now recommend the Predicting Risk of Cardiovascular Disease EVENTs (PREVENT) equations as the primary tool for estimating absolute cardiovascular risk [5]. Developed using large, diverse, and contemporary datasets, PREVENT aligns cardiovascular risk assessment with modern concepts of cardiovascular–kidney–metabolic health and enables both 10-year and 30-year risk estimation [6]. In initial external validation cohorts from the United States, PREVENT achieved C-statistics of 0.794 in women and 0.757 in men [7]. However, external validations outside the U.S. have demonstrated variable performance. For example, in a United Arab Emirates cohort, the C-index ranged from approximately 0.81–0.82 in women to 0.70–0.72 in men [8], while in a Swiss cohort of 5064 participants, the C-index ranged from 0.73 to 0.79 (76% concordant pairs), though the model systematically underestimated risk (Observed/Expected ratio ≈ 1.45) and did not outperform comparator models such as SCORE2 or PCE [9]. These findings underscore that calibration and absolute risk estimates derived from PREVENT may not translate accurately to non-U.S., ethnically diverse, or different baseline-risk populations.

The urgency for modern, more precise prediction models is particularly evident in non-European and non-American regions, where the burden of CVD is disproportionately high [10]. In middle- and low-income countries, CVD accounts for nearly a quarter of all deaths, with mortality rates at least 2.5 times higher than in high-income countries [10]. A systematic review found that widely used Western models—including Framingham, Pooled Cohort Equations, and SCORE—exhibited acceptable discriminative ability but universally poor calibration when predicting 10-year CVD risk in Asian populations [11]. This need for geographical tailoring is globally recognized, exemplified by the World Health Organization's comprehensive effort to revise its CVD risk charts, leading to 21 distinct, recalibrated models designed to better estimate risk in low- and middle-income countries [12]. Despite these large-scale efforts to adapt existing tools, the performance of risk prediction remains a challenge. While the updated SMART2 algorithm was systematically recalibrated, it still demonstrated suboptimal discrimination with a C-statistic of 0.61 (95% CI 0.53–0.68) in the Latin American subcohort [13].

Machine learning (ML) and artificial intelligence have demonstrated promising utility across cardiovascular conditions beyond AMI. In obstructive coronary artery disease, ML approaches applied to exercise-test electrocardiographic features have improved diagnostic accuracy for identifying obstructive lesions [14]. Deep learning models have shown strong performance in predicting short-term mortality in acute pulmonary embolism [15], and AI-based risk stratification has been evaluated in both coronary artery disease and atrial fibrillation [16]. Event-specific risk prediction models have also shown value in high-risk surgical populations [17]. These advances underscore that properly validated ML models can deliver clinically actionable, condition-specific risk estimates that complement and extend traditional scoring frameworks.

Furthermore, existing risk-prediction models focus on long-term horizons and composite cardiovascular outcomes pose important limitations for clinical application [4,6]. By estimating events over 10 years and combining diverse endpoints such as heart attack, stroke, heart failure, or revascularization, these models often produce aggregate risk estimates that overestimate risk and lack specificity for individual outcomes [4,6,18,19]. Emphasizing short-term, event-specific prediction is intended to enhance public health planning by enabling timely, action-oriented decision-making. Although recent models targeting specific outcomes tend to demonstrate higher accuracy [20], many still fall short due to weak methodological rigour and poor adherence to reporting standards, ultimately reducing their reliability [21]. Unlike existing tools that estimate composite cardiovascular outcomes over 10-year horizons, the present study addresses an underserved domain: short-term, event-specific AMI risk stratification using routinely collected electronic health record (EHR) data from a middle-income country setting, with transparent calibration and adherence to TRIPOD + AI guidelines [22]. The purpose of this study is to develop a model capable of independently predicting 6-month and 1-year risk of acute myocardial infarction, with exploratory extensions to 5- and 10-year horizons.

## 2 Methods

### 2.1 Study design and data source

This is a retrospective modelling study using routinely collected data from an integrated cardiovascular cohort in Colombia. The registry consolidates de-identified records of urban and rural ambulatory encounters, laboratory results, and cardiovascular procedures recorded electronically. The analytic window spanned 1990–2025, with follow-up censored at the last recorded clinical contact. The study was designed and reported in accordance with TRIPOD + AI guidance for clinical prediction models using machine learning and regression [22].

#### 2.1.1 Participants

Adults were eligible if the registry showed at least one cardiovascular risk factor, defined as any recorded diagnosis or structured field indicating hypertension, diabetes mellitus, dyslipidaemia, tobacco exposure, chronic kidney disease, chronic pulmonary disease, or obesity. Patients were not excluded based on treatment status. Although medication orders and procedures were recorded, they did not define the outcome. For time-to-event analyses, only data recorded before the event or censoring were included to prevent label leakage (i.e., to avoid incorporating post-event information into model training) Patient selection is summarized in Supplementary Fig. S1.

### 2.2 Outcomes

The analysis targeted a single endpoint: acute myocardial infarction, defined from structured fields as myocardial infarction with or without revascularization as recorded in the registry. For time-to-event modelling, time zero (baseline) was anchored at each qualifying visit (index), and event times were measured in days to the first occurrence of AMI or censoring. Primary prediction horizons were 6 months (180 days) and 1 year (365 days) to support short-term, actionable decisions. Additional horizons at 5 and 10 years were also analysed to inform operational case management and alignment with long-horizon prevention frameworks. Outcome labels were derived from structured clinical fields consistently captured across sites. No subjective adjudication was performed.

### 2.3 Predictors

Candidate predictors were selected a priori from medical literature and included: age, body mass index (BMI), LDL cholesterol, diabetes (and duration, when available), hypertension, tobacco exposure, chronic kidney disease staging, chronic pulmonary disease severity, functional mobility markers, and indicators of social vulnerability [[23], [24], [25], [26], [27], [28]]. In addition, harmonized “risk signal” from dichotomous clinical flags were synthesized using a Bayesian aggregation layer that yields a latent continuous score reflecting cumulative risk burden [29,30]. This layer takes as inputs a panel of binary clinical flags — including presence of each major risk factor, prior cardiovascular procedures, and markers of clinical surveillance gaps — and models their joint distribution via a latent Gaussian structure with empirical priors estimated on the training data. The output is a single continuous score on a logit-probability scale, static per encounter. This approach was preferred over additive comorbidity indices because it captures covariation among risk flags probabilistically, better reflecting the multiplicative nature of cardiovascular risk. Full details are in Supplementary Fig. S2 and Supplementary Table S6. All predictors were extracted from structured elements of routine care.

### 2.4 Data preparation and quality control

Variable names were harmonized using canonical text mappings to address accent and spelling variants frequent in Colombian documentation. Variables with structural missingness >80% were excluded. Implausible physiologic values were flagged by automated rules and reviewed with clinical leads. For modelling, continuous predictors were centred and scaled using parameters estimated in the training split only. Numeric missingness was imputed with training-median values; the same preprocessing pipeline was applied, unchanged, to validation and test sets to avoid information leakage. Missingness patterns and distributions were examined across sociodemographic strata, and the same preprocessing steps were applied to all subgroups. Baseline Framingham cardiovascular risk categories (when available in the registry) were retained in their original levels to enable planned concordance analyses.

### 2.5 Sample size and data partitions

The source registry comprised 3,940,059 encounter-level rows for 382,589 unique patients. Modelling was performed on patient/visit-level longitudinal tables with pre-event observations only. The AMI time-to-event model used a stratified 70%/10%/20% split (train/validation/test) on the event indicator, preserving outcome prevalence. The complementary prioritization classifier for AMI employed a 75%/25% split for development and hold-out evaluation. These partitions provide narrow uncertainty around performance and calibration estimates, given the cohort size.

### 2.6 Handling of missing data

Predictors with >80% structural missingness were excluded prior to modelling [31]. Remaining numeric missingness was imputed with training-set medians. Categorical flags were retained as recorded (including explicit “unknown” levels when present). Imputation models and scaling parameters were learned on training data and applied unchanged to validation and test [32]. Missingness indicators were evaluated as candidate features; those not improving validation AUC were excluded. Residual informative missingness is partially captured by surveillance-gap flags in the Bayesian aggregation layer; potential residual bias is acknowledged in the Limitations.

### 2.7 Model development

#### 2.7.1 Primary analysis: AMI risk classifier

To mirror clinical prioritization workflows and maximize interpretability, a shallow extreme-gradient-boosting classifier was trained on the Bayesian risk signal plus the core covariates [33]. The estimator produces calibrated probabilities interpreted as the expected 6- and 12-month AMI risk. Calibration and data preprocessing were optimized using the development subset and applied consistently to the hold-out set, with hyperparameters derived from established institutional settings. The 0.50 cut-point follows standard practice for reporting binary discrimination metrics in supervised ML for dichotomous outcomes and is an illustrative reference, not a recommended operational threshold; deployment relies on continuous probability outputs stratified into priority bands (High, Medium, Low) validated externally. Threshold-dependent metrics are reported in Supplementary Table S3–S4; time-dependent AUC and Integrated Brier Score are in Supplementary Table S5.

#### 2.7.2 Secondary analysis: complementary AMI time-to-event model

We built an interpretable-first survival ensemble for AMI combining: (i) a Cox proportional hazards model with elastic-net regularization (hazard ratios for clinical review), (ii) a random survival forest (non-linear effects and interactions), and (iii) a discrete-time logistic hazard model emphasizing early risk. Horizon-specific event probabilities (6 months, 1 year, 5 years, and 10 years) were obtained by learning convex blend weights that minimized validation Brier scores, followed by isotonic recalibration on validation data. Residual learners were permitted only if they improved validation Brier without degrading calibration. Covariates matched those of the classifier, and model fitting used pre-event records only.

#### 2.7.3 Internal validation, calibration, and leakage control

All preprocessing steps (harmonization, imputation, scaling, calibration) were estimated exclusively on training data and applied forward to validation and test sets. Blend weights and calibration layers were refit within each training/validation partition to respect uncertainty. Performance was summarized as point estimates on the held-out test sets, and sensitivity analyses computed non-parametric bootstrap intervals to quantify sampling variability for key metrics.

#### 2.7.4 Performance metrics

The AMI classifier's performance was evaluated for discrimination and calibration; threshold-dependent metrics at the 0.50 illustrative cut-point are in Supplementary Table S3. For the AMI survival model, the Brier score, C-index, and calibration-in-the-large and calibration slope were reported per horizon; time-dependent AUC and Integrated Brier Score are in Supplementary Table S5. For the AMI survival model, the Brier score, concordance-type index (C-index), and calibration-in-the-large and calibration slope were reported per horizon (6 months, 1 year, 5 years, and 10 years), and visual calibration curves were generated at 6 and 12 months. Decision-curve analyses (net benefit) were conducted on the test set to illustrate potential clinical utility across threshold probabilities pertinent to near-term AMI risk management. Consistent with a probability-as-risk framework, observed versus expected AMI events (sum of predicted probabilities) were summarized by predicted-risk strata over the test set.

#### 2.7.5 Legacy comparator and concordance analysis

To contextualize the survival-based prioritization against legacy long-horizon scores, we performed complementary concordance analyses. For each contract with complete information, we formed horizon-specific survival-priority tertiles: a short-term scheme based on the average of the 6- and 12-month survival probabilities, and a long-term scheme based on the average of the 5- and 10-year probabilities. These survival-priority tertiles were cross-tabulated with (i) baseline Framingham risk categories and (ii) tertiles of the continuous AMI-risk score derived from the classifier. Cross-tabulations and Cramér's V from chi-square tests of independence (two-sided, α = .05) were used to quantify the strength of association, with emphasis placed on effect size rather than p-values. Analyses used the subset with complete labels for each pair under comparison (N = 179,946 for the main contrasts), and detailed contingency tables are provided in the Supplement. Additional analyses using classifier-based risk tertiles as the reference scheme are also reported in the Supplement.

In a prespecified sensitivity analysis focused on a classical coronary risk score, we also computed the PROCAM 10-year coronary risk according to the standard Münster specification for men aged 35–65 years with complete data, using age, lipid profile (LDL, HDL, triglycerides), systolic blood pressure, smoking, diabetes, and family history of myocardial infarction. The resulting PROCAM_Risk_Percent (0–30%) was then compared with the model's AMI risk estimates using Pearson correlation coefficients for short-term and extended-horizon configurations, in order to quantify the degree of linear association in patient ranking between PROCAM and the Corpus probabilities. Framingham and PROCAM were chosen as legacy comparators because both have been validated and are routinely used in clinical practice in Colombia [34].

#### 2.7.6 Robustness analysis

Variable directionality (Cox coefficients) and model attribution (forest importance profiles) were inspected to ensure that no single artifact or site-specific variable dominated predictions. As a robustness check, missingness handling was varied (complete-case sensitivity), and primary endpoints were recalculated at alternate index definitions, with results remaining directionally consistent.

#### 2.7.7 External, label-delayed validation cohort

An external, label-delayed validation was conducted using a de-identified general-population cohort (n = 5602) from an independent insurance provider. Prior to scoring, the insurer's clinical committee confirmed that the cohort's age distribution was broadly comparable to the derivation population (median age 63 years vs. 64 years in the derivation cohort); detailed patient-level characteristics could not be released under the data-sharing agreement, but aggregate demographic equivalence was verified as a precondition for the validation exercise (see Supplementary Table S7). AMI probabilities were generated with the locked classifier without re-tuning. Expected events were computed as the sum of predicted probabilities and compared with subsequently observed events (O/E). Pre-specified priority bands (Low <0.25, Medium 0.25–0.75, High ≥0.75) were applied and event rates summarized per band; differences were tested by one-way ANOVA with Tukey HSD. An O/E < 1.0 in the external cohort may reflect lower baseline AMI incidence, delayed administrative coding (label-delayed design), or differential care access; recalibration to local rates is recommended before operational deployment.

#### 2.7.8 Software and reproducibility

Analyses were performed in Python 3.10; key package versions are listed in Supplementary Table S2. Version control preserved an auditable history. Reproducibility packages include scripts that regenerate all tables and figures from curated analytic files (see supplementary materials). To safeguard proprietary components, model families, covariates, and calibration procedures are reported, but full weight vectors and trained artifacts are not disclosed.

#### 2.7.9 Ethics and reporting

The study was approved by the Institutional Review Board of Salud Total with a waiver of informed consent owing to the use of de-identified, retrospective data.

#### 2.7.10 Alignment with clinical use

Focusing on 6- and 12-month AMI risk supports timely triage, targeted diagnostics (e.g., referral optimization), and proactive care management for patients at near-term risk of myocardial infarction. Modelling choices (transparent covariates, Cox-based effect summaries, strict calibration) were selected to maximize clinical interpretability and operational uptake in routine care.

## 3 Results

### 3.1 Sample characteristics

The registry comprised 382,589 patients who met eligibility criteria, contributing 3,940,059 encounters (encounter-level records). Overall, 15,511 patients experienced an acute myocardial infarction during follow-up. The patient cohort was 53.1% female and median age was 64 years [IQR 55–72]. At the encounter level, the median body mass index was 27.6 kg/m^2^ (IQR 24.9–30.9). The encounter-level median LDL cholesterol was 99 mg/dL (IQR 75–129), and LDL persisted ≥130 mg/dL in 541,065 encounters (13.7%) while triglycerides were ≥150 mg/dL in 1,180,785 encounters (30.0%). Hypertension was documented in 41.9% of encounters, diabetes in 23.7%. Among the 2,912,548 encounters with a registered smoking status, smoking was documented as active in 93,223 (3.2%) and former in 162,222 (5.6%). Chronic kidney disease stage ≥3 was recorded in 0.11% of encounters, COPD in 1.0%, and programme-level social risk indicators were present in 27.4%. Additionally, 1102 encounters (0.03%) noted a prior coronary angiography. Framingham risk classifications were assigned in 857,406 encounters, representing 21.8% of the total. Of these, 7% (about 59,000) were classified as high risk, 44% (about 377,000) as moderate risk, and 49% (about 421,000) as low risk.

### 3.2 Primary analysis: AMI risk classification

Discrimination and calibration metrics for both the classifier and the survival ensemble are summarized in Table 1. The classifier achieved an AUC of 0.869 on the held-out test set, reflecting robust rank-based discrimination. Threshold-dependent metrics are presented for illustrative purposes in Supplementary Table S3. The model's operational use relies on continuous probability outputs stratified into actionable priority bands (High, Medium, Low) intended to inform outreach scheduling, diagnostic triage, and preventive monitoring. The conventional 0.50 cut-point follows standard reporting practice for binary ML classifiers applied to dichotomous clinical outcomes [35,36] and does not constitute a recommended operational threshold.

**Table 1:** AMI classifier performance and survival prediction accuracy at 6 and 12 months, 5 years, and 10 years.

Primary Analysis							
Metric	AUC	Sensitivity	Specificity	Brier score	Precision	F1-score	Accuracy
Risk stratification performance	0.869	0.812	0.757	0.147	0.77	0.791	0.784

**Secondary Analysis**				
Survival	C-index	Brier score	Calibration intercept (95% CI)	Calibration slope (95% CI)
Survival 6 months	0.836	0.013928	−0.722 [−1.035, −0.410]	0.801 [0.714, 0.888]
Survival 12 months	0.846	0.03035	−0.628 [−0.803, −0.453]	0.761 [0.698, 0.825]
Survival 5 years [EXPLORATORY]	0.983	0.039142	0.287 [0.214–0.360]	0.685 [0.638–0.733]
Survival 10 years [EXPLORATORY]	0.993	0.020913	0.557 [0.459–0.655]	0.556 [0.525–0.586]

***Notes*.** (i) Risk stratification performance metrics characterize the model's ability to separate patients who experienced AMI from those who did not, using the predicted probability output as a ranking signal. The 0.50 probability boundary is reported as a conventional reference point — standard in supervised ML evaluation for dichotomous outcomes [18] — and serves to demonstrate that events concentrate in the high-predicted-risk group and are largely absent in the low-predicted-risk group. This is the core property required for population-level prioritization: that patients assigned high probabilities carry substantially higher observed event rates than those assigned low probabilities, as confirmed by the calibration analyses (Table 2) and external band-level validation (Table 4). These metrics are not intended as an operational decision threshold; deployment relies on continuous probability outputs stratified into priority bands. (ii) Survival metrics summarise rank discrimination (C-index), prediction error (Brier), and calibration across horizons. (iii) Rows labelled [EXPLORATORY] should not be interpreted as prospective prognostic accuracy; C-indices at 5 and 10 years reflect cumulative risk ordering within a longitudinal cohort and are reported for descriptive purposes only. Time-dependent AUC and Integrated Brier Score at actionable horizons are in Supplementary Table S5.

### 3.3 Secondary analysis: AMI time-to-event model

**Fig. 1:**
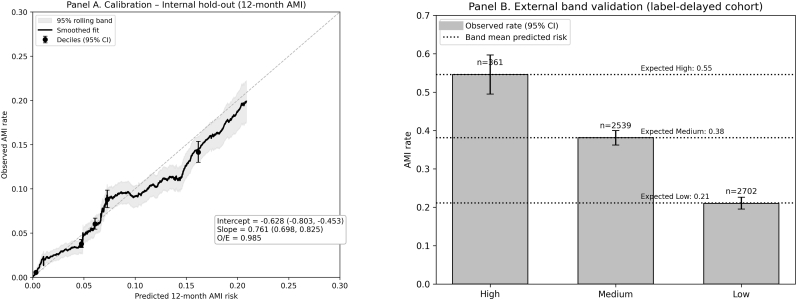
Calibration and external band validation for 6–12-month AMI risk. Panel A shows the internal hold-out calibration plot: the smoothed curve and decile-level confidence intervals closely track the 45° identity line after restricting the axes to clinically relevant risks (0–30%), with annotated intercept, slope, and O/E. Panel B displays the external, label-delayed insurer cohort: observed AMI rates (bars, 95% CIs) and the corresponding band-level mean predicted risks (horizontal dotted lines) for the High, Medium, and Low priority bands.

The complementary survival ensemble for AMI preserved low prediction error and good rank ordering at clinically actionable horizons. At 6 months, Brier = 0.013928 and C-index = 0.836; calibration-in-the-large and slope were −0.722 (95% CI −1.035 to −0.410) and 0.801 (95% CI 0.714 to 0.888), respectively. At 12 months, Brier = 0.030350 and C-index = 0.846; calibration-in-the-large and slope were −0.628 (95% CI −0.803 to −0.453) and 0.761 (95% CI 0.698 to 0.825) (see Table 1 and Fig. 1).

The negative calibration-in-the-large intercepts at short horizons indicate that the raw survival model systematically over-predicted AMI risk before isotonic recalibration was applied on the validation set. This pattern is expected in high-risk EHR cohorts where baseline hazard is elevated: the Cox component tends to over-estimate absolute risk at short horizons until recalibrated. The aggregate O/E ratio of 0.998 reflects the post-recalibration model. The calibration intercepts in Table 1 are post-isotonic-recalibration values; pre-recalibration intercepts for the survival component were not separately persisted, consistent with standard practice for ensemble models where recalibration is an integral model stage. Supplementary Note 1 provides the full technical account and deployment recommendations. The near-perfect C-indices at 5 and 10 years (0.983 and 0.993) should be interpreted with caution. These values most plausibly reflect cumulative risk ordering within a large longitudinal cohort rather than prospective prognostic accuracy: (a) informative censoring may inflate rank statistics at long horizons; (b) the maximum observed follow-up is 584 days, so 5- and 10-year estimates are extrapolations beyond the observed data range; and (c) visit frequency, correlated with risk, may act as a proxy predictor over long time spans. Time-dependent AUC and Integrated Brier Score — less susceptible to these artifacts — are reported in Supplementary Table S5.

Calibration is the central criterion for evaluating a probabilistic classifier, defined as the agreement between predicted risks and observed event frequencies. Calibration was assessed both in aggregate (the sum of predicted risks approximating observed events) and within subgroups (event rates aligning with average predicted risk after applying a threshold), results are reported in Table 2. On the hold-out test set, aggregate calibration was near perfect: observed AMI events = 4490 vs expected (sum of predicted probabilities) = 4499.3, yielding O/E = 0.998. Class-level calibration at the prespecified 0.50 threshold showed close alignment between observed proportions and average predicted risks: for Predicted 0 (n = 3652), the observed AMI rate was 16.1% (95% CI 14.9–17.3) versus an average predicted risk of 17.4% (Expected = 636.6; Obs−Exp = −49.6); for Predicted 1 (n = 5328), the observed AMI rate was 73.3% (95% CI 72.1–74.5) versus an average predicted risk of 72.5% (Expected = 3862.7; Obs−Exp = +40.3). The small, opposite-signed differences and overlap between observed rates and average predicted risks indicate probability coherence at the operational cut-point.

**Table 2:** Observed vs. Expected AMI events by predicted class.

Predicted class	Patients	Observed AMI, n (%)	Expected AMI, n	Obs − Exp	95% CI for observed proportion
Class of low expected AMI	3652	587 (16.1%)	636.6	−49.6	14.9%–17.3%
Class of high expected AMI	5328	3903 (73.3%)	3862.70	40.3	72.1%–74.5%

### 3.4 Concordance with horizon-specific risk strata

Horizon-specific survival priorities demonstrated moderate alignment with legacy risk frameworks. Concordance statistics, cross-tabulations with Framingham categories and continuous AMI-risk tertiles, and correlations with PROCAM estimates are summarized in Table 3. Moderate concordance with Framingham (Cramér's V = 0.326 at short horizons) is both expected and operationally desirable: the two approaches measure fundamentally different constructs. Framingham estimates 10-year absolute risk based on stable chronic risk-factor burden, whereas the present model captures near-term event probability integrating dynamic clinical signals — biomarker trajectories, comorbidity accrual, and surveillance gaps — invisible to a static scoring equation. A patient classified as moderate 10-year risk by Framingham may carry substantially elevated 6-month AMI probability if glycaemic control has recently deteriorated, renal function has declined, or clinical contact has been interrupted. This is precisely the added operational value of the present model. An illustrative example is in Supplementary Box 1. The two frameworks are therefore complementary: Framingham supports long-horizon prevention planning while the present model enables near-term prioritization of outreach and diagnostics.

**Table 3:** Horizon-specific cross-classification of survival priorities, Framingham risk groups, and continuous AMI-risk tertiles (row-wise %).

Horizon	Comparator scheme	Reference stratum	High	Medium	Low
6–12 months	Framingham group	Survival priority: Low	75.32	19.14	5.54
6–12 months	Framingham group	Survival priority: Medium	61.49	23.67	14.84
6–12 months	Framingham group	Survival priority: High	26.98	26.10	46.93
6–12 months	Survival priority	Risk tertile: Low	20.45	37.26	42.29
6–12 months	Survival priority	Risk tertile: Medium	27.84	37.30	34.86
6–12 months	Survival priority	Risk tertile: High	41.51	35.07	23.42
5–10 years	Framingham group	Survival priority: Low	71.46	19.96	8.58
5–10 years	Framingham group	Survival priority: Medium	59.01	23.19	17.80
5–10 years	Framingham group	Survival priority: High	31.71	26.24	42.05
5–10 years	Survival priority	Risk tertile: Low	16.50	35.83	47.67
5–10 years	Survival priority	Risk tertile: Medium	34.36	33.65	31.99
5–10 years	Survival priority	Risk tertile: High	49.10	30.53	20.37

Notes. Cramér's V: 0.326 (short survival vs. Framingham), 0.148 (short survival vs. continuous-risk tertiles), 0.266 (long survival vs. Framingham), 0.215 (long survival vs. continuous-risk tertiles). N = 179,946 contracts with both measures available. Moderate discordance with Framingham at short horizons is expected given the different risk constructs measured; see text for interpretation and Supplementary Box 1 for an illustrative example.

As a complementary legacy comparison, we also examined concordance with the PROCAM 10-year coronary risk score in the subset of men aged 35–65 years with complete data. PROCAM_Risk_Percent was significantly correlated with the model's AMI risk estimates, but only with low to moderate magnitude: r = 0.24 (n = 44,045 pairs) for the short-horizon configuration and r = 0.34 (n = 53,557 pairs) for the extended-horizon configuration (both p < .001). These results indicate a statistically significant but only partial alignment in patient ranking between PROCAM and the Corpus AMI probabilities.

### 3.5 External, label-delayed validation

The model predicted 1736 AMI events (sum of probabilities), while the observed count was 1,370, yielding O/E = 0.79. This degree of over-prediction may reflect lower baseline AMI incidence in the validation population, delayed administrative coding of events (label-delayed design), or differential access to acute care; it does not necessarily indicate model miscalibration, and recalibration to local event rates is recommended before deployment in any new health system. Priority bands showed monotonic separation of event rates, and ANOVA confirmed highly significant between-group differences (F = 147.6; p < .001); Tukey HSD indicated that all pairwise contrasts were significant (p < .001). Band-level event rates with 95% CIs are reported in Fig. 1 and in Table 4.

**Table 4:** External label-delayed validation: overall observed vs expected and event rates by priority band.

Group	N	Observed AMI	n (%), 95% CI	*p*
Overall	5602	1370 (24.5%)	23.3%–25.7%	<0.001
High (≥0.75)	361	197 (54.6%)	49.5%–59.7%	<0.001
Medium (0.25–0.75)	2539	967 (38.1%)	36.2%–40.0%	<0.001
Low (<0.25)	2702	567 (21.0%)	19.5%–22.6%	<0.001

*Notes.* (i) Expected AMI for the overall row is the sum of predicted probabilities from the locked classifier; band-level expected counts were not required for the ANOVA/Tukey procedure and are therefore omitted. (ii) 95% CIs are binomial (approximate). (iii) Minor tally differences can arise from timing/definition reconciliation between global and band-specific tallies; conclusions are unchanged (monotonic separation and significant between-group differences).

## 4 Discussion

Current clinical decision-making remains limited by the imprecision of existing cardiovascular disease CVD risk models, indicating that new methods are necessary to facilitate better clinical guidance. For instance, a comparison of the ACC/AHA, Adult Treatment Panel III, and European Society of Cardiology guidelines found that all three models provided poor calibration, underscoring the necessity of improving risk predictions and setting appropriate population-wide thresholds [37]. In this context, the future of CVD prevention is increasingly seen to rely on the adoption of advanced methods like machine learning and digital health technology. Machine learning is capable of generating insights from complex, big data derived from electronic health records, imaging, wearables, and sensors, which can drive a shift toward personalized, precision CVD prevention [38].

In this study, we evaluated a probabilistic model developed to estimate the short-term risk of AMI over 6- and 12-month horizons in a large, real-world population. Our aim was to characterize the model's discriminatory capacity, calibration, and prediction error, and to assess how the resulting prioritization structure aligns with established long-horizon cardiovascular risk classifications. The findings show that near-term AMI risk can be estimated with high accuracy using routinely collected electronic health record data, and that primary 6- and 12-month horizons demonstrate consistent discrimination and calibration. Extended 5- and 10-year horizon analyses are reported as exploratory; the near-perfect C-indices at those horizons most plausibly reflect cumulative risk ordering within a longitudinal cohort — amplified by informative censoring, visit-frequency correlation with risk, and extrapolation beyond the observed 584-day follow-up window — rather than prospective prognostic accuracy. Calendar-time effects and variable completeness of historical look-back diagnoses may contribute additional non-stationarity, reinforcing the need for ongoing monitoring and recalibration. Discrimination was high across components, with an AUC of 0.869 for the classifier and C-indices of 0.836 and 0.846 for the 6- and 12-month survival models, respectively. The calibration slopes (0.76–0.80) confirmed strong risk separability, while the calibration intercepts, after isotonic recalibration, confirmed that aggregate predicted event counts closely matched observed events at the population level (O/E = 0.998). Brier scores indicated low prediction error, and calibration assessments showed close agreement between predicted probabilities and observed event frequencies. Differences between observed and expected events were small in both aggregate and threshold-based subgroup analyses, indicating that the model's output probabilities functioned as reliable estimates of AMI risk.

This model provides clinicians with an event-specific tool capable of estimating AMI risk in a way that is conceptually comparable to traditional long-horizon risk scores. In Colombia, Framingham has demonstrated poor discrimination (AUC 0.65) and consistent overestimation of 10-year risk, while PROCAM—although better calibrated—has shown only modest discrimination (AUC 0.74) and miscalibration in high-risk groups [34]. In contrast, our model delivers materially higher discrimination and stronger calibration, suggesting that it may offer more clinically meaningful estimates of imminent coronary risk. However, because our model was not derived from a general population cohort, further external validation remains necessary. This process is currently underway.

Second, the model may have its greatest utility as a population-health tool. Health systems increasingly need mechanisms to stratify cardiovascular risk within large, heterogeneous populations. By providing calibrated, near-term probabilities rather than broad 10-year composite categories, the model supports more precise prioritization of care pathways, enabling earlier diagnostic evaluation, structured follow-up, and targeted preventive interventions for individuals at highest imminent risk [39]. These advantages are especially relevant in middle- and low-income countries, where diagnostic capacity is often constrained [40]. The probabilistic nature of the model also facilitates clearer risk communication and more informed resource planning, which may strengthen population-level initiatives aimed at reducing avoidable acute events, guiding disease-management outreach, and allocating preventive resources according to anticipated near-term burden [38].

A growing body of work has explored routinely collected data and advanced analytic methods for cardiovascular risk stratification, with important differences in purpose and time horizon. Population-oriented tools have primarily focused on long-term absolute risk for prevention planning, while post-AMI prognostic models have emphasised longer-term mortality or composite outcomes rather than near-term prioritization [41]. Evaluations of models embedded in clinical guidelines have highlighted that most are designed around long-horizon endpoints and show substantial methodological limitations, including limited calibration assessment across settings [21]. The present study contributes by focusing explicitly on short-term AMI risk as a population-health stratification problem, emphasizing risk separation, calibration, and operational interpretability. Future work should assess the generalizability of these findings in external cohorts and across diverse demographic and socioeconomic settings. Prospective evaluation will be necessary to determine whether integrating short-term AMI predictions into clinical workflows improves care quality, diagnostic yield, or patient outcomes [42,43].

### 4.1 Operational considerations

Deployment at population scale requires ongoing lifecycle management. Calibration should be monitored using rolling O/E ratios at regular intervals (e.g., quarterly); a drift of ±20% from the reference O/E should trigger recalibration — updating the isotonic correction layer on recent data without retraining the base model — with full retraining reserved for evidence of distributional shift. Governance responsibilities should be clearly assigned, including an accountable clinical lead and a defined escalation pathway. Probabilistic outputs should be communicated to non-technical stakeholders as priority bands (High, Medium, Low) with observed event rates from validation data, rather than raw probabilities, to support operational uptake without inducing disproportionate alarm.

### 4.2 Limitations

The study was conducted within a single health system, and performance may differ in populations with different demographic characteristics, care-seeking patterns, or prevalence of cardiovascular disease. As with all EHR-based studies, the model's accuracy depends on the completeness and accuracy of coded data; underdiagnosis, miscoding, and irregular care utilization may introduce misclassification. We did not evaluate potential temporal drift, and model performance may change over time as clinical practices evolve. Missing data in EHR settings may be non-random; median imputation may inadequately capture informative absence, particularly for laboratory values, and could introduce bias. Although missingness indicators were evaluated during model development and partially captured through the Bayesian aggregation layer's surveillance-gap flags, residual bias from informative missingness cannot be excluded. The survival ensemble employs cause-specific censoring, treating non-AMI deaths as censored observations. In older populations with elevated competing mortality, this approach may systematically overestimate AMI risk. Cause-specific modelling was selected to maintain alignment with AMI-specific prioritization objectives, and competing risk extensions using Fine–Gray models are planned for future work. The near-perfect C-indices at 5 and 10 years (0.983 and 0.993) should not be interpreted as prospective prognostic accuracy; they most plausibly reflect artefactual rank separation due to informative censoring and extrapolation beyond the observed follow-up window (maximum 584 days). Recalibration to local event rates is required before deployment in any new health-system context, as demonstrated by the O/E = 0.79 in the external validation cohort.

This study does not differentiate between types of acute myocardial infarction, such as ST-segment elevation myocardial infarction and non-ST-segment elevation myocardial infarction, which may affect risk stratification in specific populations. Further methodological development may enhance performance. Incorporating longitudinal trajectories, natural-language data from clinical notes, or multimodal inputs such as electrocardiographic features could further refine risk estimation. Finally, research on risk communication is warranted to guide how short-term probabilities can be presented to clinicians and patients in a manner that supports decision-making without inducing alarm or overuse of diagnostic resources.

## 5 Conclusions

In a large outpatient population, a probabilistic model using routinely collected electronic health record data enabled stratification by estimated 6- and 12-month risk of acute myocardial infarction, with consistent discrimination and calibration at the population level. These findings highlight the potential role of short-term, event-specific risk estimation as a complement to traditional long-horizon cardiovascular risk frameworks, particularly for supporting prioritization and planning within health systems. As a population-health stratification tool, this approach may inform the allocation of diagnostic, monitoring, and preventive resources according to anticipated near-term burden. Recalibration to local incidence rates is recommended before deployment in new health-system contexts. Further evaluation in real-world operational settings is needed to assess its potential to reduce avoidable acute events and improve cardiovascular outcomes.

## Availability of data and materials

Data may be made available upon reasonable request to Juan Esteban Correa-Morales.

## Declaration of generative AI and AI-assisted technologies in the manuscript preparation process

During the preparation of this work the author(s) used Calude Sonnet 4.6 in order to check gramar and text readibility. After using this tool/service, the author(s) reviewed and edited the content as needed and take(s) full responsibility for the content of the published article.

## Funding

This study received no external funding.

## CRediT authorship contribution statement

**Jose David Amorocho-Morales:** Conceptualization, Data curation, Formal analysis, Funding acquisition, Investigation, Methodology, Resources, Visualization, Writing – original draft. **Sergio Parra Guevara:** Data curation, Investigation, Methodology, Validation, Writing – review & editing. **Elias Quintero-Muñoz:** Data curation, Investigation, Supervision, Validation, Writing – review & editing. **Guillermo Dimas:** Conceptualization, Investigation, Methodology, Project administration, Resources, Supervision, Validation, Writing – review & editing. **Juan Esteban Correa-Morales:** Conceptualization, Formal analysis, Investigation, Methodology, Resources, Validation, Writing – original draft, Writing – review & editing.

## Declaration of competing interest

José David Amorocho-Morales and Juan Esteban Correa-Morales are employees, co-founders, and shareholders of CorpusAI, the company that developed the algorithm evaluated in this study. The remaining authors declare no known competing financial interests or personal relationships that could have appeared to influence the work reported in this paper.
