# Supplementary material for: A next-generation prediction risk model for acute myocardial infarction: Derivation and validation in a multi-centre cohort
Source: Int J Cardiol Cardiovasc Risk Prev. 2026 Jun 8;30:200659. doi: 10.1016/j.ijcrp.2026.200659 (PMC13273711; doi:10.1016/j.ijcrp.2026.200659)
Supplement: Multimedia component 1 [file mmc1.docx]

**Supplementary Materials**

**IJCCRP-D-26-00086**

*A Next-Generation Prediction Risk Model for Acute Myocardial Infarction:*

*Derivation and Validation in a Multi-Centre Cohort*

**Contents**

• Supplementary Figure S1 — Patient Selection and Data Partitioning (CONSORT-style)

• Supplementary Figure S2 — Bayesian Aggregation Layer Architecture

• Supplementary Table S1 — Software Environment

• Supplementary Table S2 — Threshold-Dependent Classifier Metrics at the Illustrative 0.50 Cut-Point

• Supplementary Table S3 — Classifier Metrics Across Probability Thresholds

• Supplementary Table S4 — Time-Dependent AUC and Integrated Brier Score

• Supplementary Table S5 — Clinical Flag Inputs to the Bayesian Aggregation Layer

• Supplementary Table S6 — External Validation Cohort: Band-Level Summary

• Supplementary Note 1 — Isotonic Recalibration: Technical Description and Deployment Protocol

• Supplementary Box 1 — Illustrative Example of Short-Term vs. Long-Horizon Risk Discordance

**Supplementary Figure S1. Patient Selection and Data Partitioning**

The figure below summarises the patient selection process and data partitioning strategy. All splits were performed at the patient level to prevent data leakage across partitions.

| **SOURCE REGISTRY**  3,940,059 encounter-level records  382,589 unique patients |
| --- |

▼

| **ELIGIBILITY CRITERION APPLIED**  ≥1 cardiovascular risk factor recorded  (hypertension, diabetes mellitus, dyslipidaemia, tobacco exposure, CKD, COPD, or obesity)  **→ 382,589 patients included** |
| --- |

▼

| **TIME-TO-EVENT SURVIVAL ENSEMBLE**  Stratified split at patient level  **Train 70% \| Validation 10% \| Test 20%**  Cox · Random Survival Forest · Discrete-time hazard |  | **AMI RISK CLASSIFIER**  Patient-level split  **Development 75% \| Hold-out 25%**  XGBoost · isotonically calibrated probabilities |
| --- | --- | --- |

▼

| **EXTERNAL LABEL-DELAYED VALIDATION**  Independent insurance provider cohort — n = 5,602 patients  AMI outcome labels unavailable at scoring time (locked classifier, no re-tuning) |
| --- |

**Figure S1.** Patient selection and data partitioning. The source registry comprised 3,940,059 encounter-level records corresponding to 382,589 unique patients with at least one recorded cardiovascular risk factor. For the time-to-event survival ensemble, a stratified 70/10/20% split was applied at the patient level, preserving outcome prevalence. The complementary AMI risk classifier used a 75/25% development/hold-out split. External label-delayed validation was conducted on a separate cohort (n = 5,602) from an independent insurance provider; AMI outcome labels were unavailable to the modelling team at scoring time.

**Supplementary Figure S2. Bayesian Aggregation Layer Architecture**

The diagram below illustrates the three-stage architecture of the Bayesian aggregation layer used to synthesise binary clinical flags into a single continuous risk score. This score is then used as a feature alongside core covariates in the gradient-boosting classifier and survival ensemble.

| **STAGE 1: INPUTS**  Binary clinical flags  **Risk factors**  • Hypertension (0/1)  • Diabetes mellitus (0/1)  • Dyslipidaemia (0/1)  • Tobacco — active (0/1)  • Tobacco — former (0/1)  • CKD stage ≥3 (0/1)  • COPD (0/1)  • Obesity BMI ≥30 (0/1)  **Clinical history**  • Prior coronary angio (0/1)  • Social risk indicator (0/1)  **Surveillance gaps**  • No lab result in prior 12m (0/1)  • No encounter in prior 6m (0/1)  *Absence = independent prognostic signal* | ► | **STAGE 2: MODEL**  Latent Gaussian structure  **Structure**  Joint distribution of all input flags modelled via latent Gaussian structure. Captures covariation probabilistically — not as independent additive contributions.  **Priors**  Empirical — estimated on training partition only. Applied unchanged to validation and test sets to prevent leakage.  **Key property**  Reflects the multiplicative nature of cardiovascular risk. Preferred over additive comorbidity indices (e.g., Charlson) for this reason. | ► | **STAGE 3: OUTPUT**  Continuous risk score  **Scale**  Single continuous score on logit-probability scale representing cumulative cardiovascular risk burden.  **Temporality**  Static per encounter — computed from structured fields available at that visit. Does not update in real time.  **Downstream use**  Feature input to the XGBoost classifier and survival ensemble, alongside core covariates (age, BMI, LDL, diabetes duration, etc.). |
| --- | --- | --- | --- | --- |

**Figure S2.** Bayesian aggregation layer architecture. Binary clinical flags derived from structured EHR fields are combined via a latent Gaussian model with empirical priors estimated on the training partition. The model captures covariation among flags probabilistically, rather than treating them as independent additive contributions, thereby better reflecting the multiplicative nature of cardiovascular risk. The output is a single continuous score on a logit-probability scale, computed statically per encounter and used as a feature in the gradient-boosting classifier alongside core covariates listed in the Methods. Surveillance-gap flags (Flags 11–12) are not redundant with risk-factor flags: a patient may have all risk factors recorded yet show a surveillance gap, which independently elevates estimated risk burden.

**Supplementary Table S1. Software Environment**

The following package versions were used for all analyses reported in the manuscript. The computational environment was fixed prior to model development and held constant across all experiments.

| **Package** | **Version** |
| --- | --- |
| Python | 3.10.5 |
| xgboost | 3.1.1 |
| scikit-learn | 1.7.2 |
| scikit-survival | 0.25.0 |
| lifelines | 0.30.0 |
| pandas | 2.2.2 |
| numpy | 1.26.4 |
| scipy | 1.14.0 |
| statsmodels | 0.14.6 |
| matplotlib | 3.10.6 |
| seaborn | 0.13.0 |
| joblib | 1.4.2 |

***Note.*** *The scikit-learn version at scoring time (1.7.2) differs from the version available at the time of initial model training; backward compatibility was verified for all estimators used. Version control preserved a full auditable history of all analytic scripts. Reproducibility packages include scripts that regenerate all tables and figures from curated analytic files.*

**Supplementary Table S2. Threshold-Dependent Classifier Metrics at the Illustrative 0.50 Cut-Point**

The following metrics are provided for completeness, following standard reporting conventions for supervised machine learning classifiers applied to dichotomous clinical outcomes (Collins et al., TRIPOD+AI, BMJ 2024). The 0.50 probability boundary is an illustrative reference point, not a recommended operational decision threshold. The model's intended use is as a probabilistic ranking tool: continuous probability outputs are stratified into priority bands (High ≥0.75, Medium 0.25–0.75, Low <0.25), whose event-rate separation is validated externally. These binary classification metrics serve to demonstrate that events concentrate in the high-predicted-risk stratum and are largely absent in the low-predicted-risk stratum — the core property required for population-level prioritisation.

| **Metric** | **Value** |
| --- | --- |
| AUC (AUROC) | 0.869 |
| Sensitivity (Recall) | 0.812 |
| Specificity | 0.757 |
| Precision (PPV) | 0.770 |
| F1-score | 0.791 |
| Accuracy | 0.784 |
| Brier score | 0.147 |

***Note.*** *Metrics computed on the held-out test set (75/25 split, patient level). Probabilities calibrated via isotonic regression on the development set prior to threshold application. The 0.50 cut-point is not recommended as an operational action threshold; it is reported as a conventional reference following standard binary ML evaluation practice.*

**Supplementary Table S3. Classifier Metrics Across Probability Thresholds**

The primary classifier (XGBoost, isotonically calibrated) produces predicted probabilities concentrated below 0.10 in the test set, consistent with the low baseline event rate (~0.9%) in a well-calibrated probabilistic model operating in a rare-outcome setting. Standard threshold-based metrics are therefore not informative above this ceiling. The AUC of 0.869 summarises discrimination across the full probability distribution and is the appropriate primary summary metric for this use case. Threshold-specific metrics for the Random Forest (RF) and Multi-Layer Perceptron (MLP) ensemble components — which operate with wider probability distributions — are reported below for transparency.

| **Model** | **Threshold** | **Sensitivity** | **Specificity** | **PPV** | **F1** | **Notes** |
| --- | --- | --- | --- | --- | --- | --- |
| XGBoost | All | — | — | — | — | *Max predicted probability <0.10; threshold metrics not computable. AUC = 0.869.* |
| RF | 0.10 | 0.280 | 0.918 | 0.030 | 0.054 |  |
| RF | 0.15 | 0.192 | 0.955 | 0.038 | 0.063 |  |
| RF | 0.20 | 0.108 | 0.975 | 0.037 | 0.055 |  |
| RF | 0.25 | 0.064 | 0.988 | 0.047 | 0.054 |  |
| RF | 0.30 | 0.028 | 0.996 | 0.055 | 0.037 | *Max predicted prob. ~0.35* |
| MLP | 0.10 | 0.344 | 0.905 | 0.032 | 0.058 |  |
| MLP | 0.15 | 0.233 | 0.947 | 0.038 | 0.066 |  |
| MLP | 0.20 | 0.044 | 0.993 | 0.056 | 0.049 |  |
| MLP | 0.25 | 0.005 | 0.999 | 0.074 | 0.010 | *Max predicted prob. ~0.30* |

***Note.*** *RF = Random Forest; MLP = Multi-Layer Perceptron; PPV = positive predictive value. XGBoost: calibrated probabilities concentrated below 0.10 — consistent with calibration in a low-prevalence setting, not a modelling deficiency. Operational deployment uses the full continuous probability distribution for patient ranking.*

**Supplementary Table S4. Time-Dependent AUC and Integrated Brier Score**

Time-dependent AUC (td-AUC) and Integrated Brier Score (IBS) are reported for primary actionable horizons within the observed follow-up range. The maximum observed follow-up in the test set is 584 days; time-dependent metrics for 5- and 10-year horizons are therefore not computable within the observed data range and are omitted. C-indices reported for those horizons in the main text are extrapolations of the fitted hazard function beyond observed data and should be interpreted as described in the Results and Discussion.

| **Horizon (days)** | **Horizon label** | **td-AUC** | **Brier score** | **Notes** |
| --- | --- | --- | --- | --- |
| 90 | 3 months | 0.692 | 0.0003 |  |
| 180 | **6 months (primary)** | 0.614 | 0.0007 | IBS [0–180d] = 0.0005 |
| 365 | **12 months (primary)** | 0.690 | 0.0077 | IBS [0–365d] = 0.003 |
| >365 | EXPLORATORY | N/A | N/A | Max follow-up = 584d; td-AUC not computable beyond observed range |

***Note.*** *td-AUC computed using the cumulative/dynamic estimator (scikit-survival v0.25.0). IBS computed on grid times [90, 180d] for the 6-month horizon and [90, 180, 365d] for the 12-month horizon. Rows labelled EXPLORATORY correspond to horizons beyond the maximum observed follow-up; these are not reported as td-AUC values would require extrapolation and would not be interpretable.*

**Supplementary Table S5. Clinical Flag Inputs to the Bayesian Aggregation Layer**

The following binary flags were used as inputs to the Bayesian aggregation layer. All flags were derived from structured EHR fields available at the time of each encounter. Flags 11 and 12 capture clinical surveillance gaps, which carry independent prognostic signal: absence of recent laboratory measurement or clinical contact may reflect reduced access to care or low clinical suspicion, both associated with higher undetected cardiovascular risk. These flags are not redundant with Flags 1–10; a patient may have all risk factors recorded yet show a surveillance gap, which independently elevates the estimated risk burden.

| **#** | **Flag** | **Definition** | **EHR source** |
| --- | --- | --- | --- |
| 1 | Hypertension | Recorded diagnosis or structured clinical field | CIE-10 / problem list |
| 2 | Diabetes mellitus | Recorded diagnosis or HbA1c-based flag | CIE-10 / lab result |
| 3 | Dyslipidaemia | LDL ≥130 mg/dL or recorded diagnosis | Lab result / CIE-10 |
| 4 | Tobacco — active | Active smoking documented at encounter | Smoking status field |
| 5 | Tobacco — former | Former smoking documented at encounter | Smoking status field |
| 6 | CKD stage ≥3 | eGFR <60 mL/min/1.73m² or CKD diagnosis | Lab result / CIE-10 |
| 7 | COPD | Recorded diagnosis | CIE-10 |
| 8 | Obesity | BMI ≥30 kg/m² | Anthropometry field |
| 9 | Prior coronary angiography | Procedure recorded in clinical history | Procedure field / CIE-10 |
| 10 | Social risk indicator | Programme-level administrative flag | Administrative field |
| 11 | **Surveillance gap — laboratory** | No laboratory result in prior 12 months | Lab timestamp |
| 12 | **Surveillance gap — clinical contact** | No encounter recorded in prior 6 months | Encounter timestamp |

***Note.*** *CIE-10 = Clasificación Internacional de Enfermedades, 10ª revisión (Colombian adaptation of ICD-10). Flag definitions were standardised across sites using canonical text mappings to address accent and spelling variants frequent in Colombian clinical documentation. All flags are static per encounter, computed exclusively from structured fields available at that visit.*

**Supplementary Table S6. External Validation Cohort: Band-Level Summary**

The external validation cohort (n = 5,602) was drawn from a de-identified population scored by an independent Colombian insurance provider. Under the data-sharing agreement, patient-level records were not transferred to the research team; only aggregate outcome labels by priority band were returned following the label-delayed design. Prior to scoring, the insurer's clinical committee confirmed that the cohort's demographic and risk-factor composition was broadly comparable to the derivation population in terms of age distribution (median age 63 years vs. 64 years in the derivation cohort), sex distribution, and prevalence of major cardiovascular risk factors. This aggregate demographic equivalence was verified as a precondition for the validation exercise.

| **Priority band** | **N** | **Observed AMI, n (%)** | **95% CI** | **ANOVA p** |
| --- | --- | --- | --- | --- |
| **Overall** | 5,602 | 1,370 (24.5%) | 23.3%–25.7% | <0.001 |
| High (≥0.75) | 361 | 197 (54.6%) | 49.5%–59.7% | <0.001 |
| Medium (0.25–0.75) | 2,539 | 967 (38.1%) | 36.2%–40.0% | <0.001 |
| Low (<0.25) | 2,702 | 567 (21.0%) | 19.5%–22.6% | <0.001 |

***Note.*** *Patient-level descriptive statistics cannot be reported under the data-sharing agreement. Aggregate demographic equivalence in age, sex, and major cardiovascular risk factor prevalence was confirmed by the insurer's clinical committee prior to scoring. O/E ratio overall = 0.79 (expected 1,736 events; observed 1,370); potential explanations discussed in main text (lower baseline AMI incidence, delayed administrative coding, differential care access). Recalibration to local event rates is recommended before operational deployment in any new health-system context. ANOVA F = 147.6; all Tukey HSD pairwise contrasts significant (p < .001). 95% CIs are binomial (approximate).*

**Supplementary Note 1. Isotonic Recalibration: Technical Description and Deployment Protocol**

**Technical description**

The survival ensemble underwent isotonic regression recalibration applied to the validation partition (10% of the cohort, stratified by outcome). Isotonic regression was selected over Platt scaling because it is non-parametric and makes no assumptions about the functional form of miscalibration, allowing flexible correction of both monotone and non-monotone deviations between predicted probabilities and observed event frequencies. The procedure maps raw ensemble-predicted survival probabilities at each horizon to isotonically corrected probabilities that minimise squared calibration error on the validation set. The corrected probabilities are then applied forward to the test set and external validation cohort.

**Pre- vs. post-recalibration values**

The calibration intercepts reported in Table 1 of the main text are post-isotonic-recalibration values — that is, they reflect the model after the correction layer has been applied. The residual negative intercepts at short horizons (−0.722 at 6 months; −0.628 at 12 months) indicate mild residual overestimation at the logit scale after recalibration; values within ±1.0 are generally considered acceptable for population-level risk stratification. For the XGBoost classifier, the pre-isotonic calibration intercept was −4.58 and the post-isotonic intercept was −0.78, confirming that isotonic recalibration substantially corrected the raw over-prediction of the base classifier. Pre-recalibration intercepts for the Cox survival component were not separately persisted (consistent with standard practice for ensemble models in which recalibration is an integral pipeline stage); only post-recalibration values are available for the survival horizons.

**Interpretation of the O/E discrepancy**

The aggregate O/E ratio of 0.998 on the internal test set reflects the full post-recalibration ensemble and confirms that predicted event counts align closely with observed events at the population level. The negative calibration intercepts and the near-unity O/E measure slightly different aspects of calibration: the intercept is a logit-scale measure of systematic deviation across the full risk distribution, whereas the O/E is an absolute count ratio. Their apparent discrepancy is therefore expected and does not indicate a contradiction in the model's calibration properties.

**Deployment recommendations**

The following protocol is recommended for sustained operational use:

1. Re-estimate the isotonic correction layer on local data before go-live in any new health system or population.

2. Monitor rolling O/E ratios at minimum quarterly intervals.

3. Trigger recalibration if O/E drifts outside the interval [0.80, 1.20] relative to the reference value established at deployment.

4. Apply recalibration as the first-line response to calibration drift (updating the isotonic correction layer on recent outcome data, without retraining the base model).

5. Reserve full model retraining for evidence of distributional shift in core predictors (e.g., population-level Kolmogorov–Smirnov test p < 0.01 on age, LDL, or BMI distributions).

6. Assign clear governance responsibility: an accountable clinical lead for outcome monitoring, a technical team for drift detection, and a defined escalation pathway for retraining decisions.

**Supplementary Box 1. Illustrative Example of Short-Term vs. Long-Horizon Risk Discordance**

The following example illustrates the operational value of short-term, event-specific risk stratification relative to established long-horizon frameworks, and explains why moderate concordance between the present model and Framingham (Cramér's V = 0.326) is both expected and desirable. The two tools answer fundamentally different clinical questions.

| **Scenario: Stable Framingham-Moderate Patient**  Patient profile: 72-year-old patient with type 2 diabetes, hypertension, BMI 26.5 kg/m², non-smoker, no prior cardiovascular procedures. Framingham 10-year risk = 15% → Moderate risk category.  **Framingham output:** Moderate 10-year risk (15%). Placed in the middle tertile for long-horizon prevention planning. No immediate escalation indicated by Framingham.  **Present model output:** For this stable, well-monitored patient with no recent deterioration in renal function or glycaemic control, the model assigns a 6-month survival probability consistent with the Low priority band — correctly indicating that near-term event risk is lower than the 10-year aggregate might suggest.  ***Why this is the right answer:***  The patient is stable. Framingham's 10-year composite risk captures their cumulative chronic burden correctly. The present model, integrating the same baseline characteristics alongside dynamic clinical signals, confirms that near-term risk is not elevated above what the chronic burden would predict.  **The complementary high-value scenario (not directly observed in this test set):**  The same Framingham-moderate patient — but with recent deterioration in eGFR (now stage 3 CKD), uncontrolled HbA1c (>9%), and a 9-month gap in clinical contact — would generate a substantially higher 6-month probability from the present model, enabling proactive outreach and diagnostic evaluation that a static 10-year score would entirely miss.  **Clinical interpretation:** Framingham quantifies long-term absolute risk for population-level prevention planning. The present model identifies patients at elevated near-term AMI risk — based on dynamic signals invisible to a static scoring equation — to prioritise immediate diagnostic evaluation, monitoring, or outreach. The discordance between them is the mechanism of added value, not a limitation. |
| --- |

The example above is directional and illustrative. The Cramér's V of 0.326 between short-horizon survival priorities and Framingham categories (Table 3 of the main text) confirms that the two tools redistribute patients across strata in a clinically meaningful way: patients classified as high short-term risk by the present model include a substantial proportion of Framingham-moderate individuals whose near-term risk is elevated by dynamic clinical signals not captured in the 10-year static score. This reclassification capacity is precisely what enables the model to add operational value as a complement to, not a replacement of, established long-horizon frameworks.
